# Salivary microbiome in chronic kidney disease: what is its connection to diabetes, hypertension, and immunity?

**DOI:** 10.1186/s12967-022-03602-5

**Published:** 2022-09-04

**Authors:** Fengping Liu, Jiayi Sheng, Lei Hu, Bin Zhang, Wei Guo, Yang Wang, Yifeng Gu, Peng Jiang, Hao Lin, Brako Lydia, Yifan Sun, Yifan Tang, Chaoqun Gu, Shichao Wei, Qixiao Zhai, Weiguo Chen, Ninghan Feng

**Affiliations:** 1 Wuxi School of Medicine, Jiangnan University, Wuxi, 214122 Jiangsu China; 2 Department of Urology, Wuxi No.2 People’s Hospital, Affiliated Wuxi Second Hospital of Nanjing Medical University, Wuxi, 214000 Jiangsu China; 3 Department of Urology, the First Affiliated Hospital of Soochow University, Suzhou, 215006 Jiangsu China; 4 Collaborative Innovation Center for Diagnosis and Treatment of Infectious Diseases, State Key Laboratory for Diagnosis and Treatment of Infectious Diseases, School of Medicine, The First Affiliated Hospital, Zhejiang University, Hangzhou, 310003 Zhejiang China; 5 School of Medicine, Nantong University, Nantong, 226019 Jiangsu China; 6 State Key Laboratory of Food Science and Technology and School of Food Science and Technology, Jiangnan University, Wuxi, 214122 China

**Keywords:** Chronic kidney disease, Diabetes, Hypertension, Immunology, Salivary microbiome

## Abstract

**Background:**

The association between oral dysbiosis and chronic kidney disease (CKD) has gained increasing attention in recent years. Diabetes and hypertension are the most common conditions in CKD. However, a case–control study with matched confounding variables on the salivary microbiome in CKD and the influence of diabetes and hypertension on the microbiome has never been reported.

**Methods:**

In our study, we compared the salivary microbiome profile between patients with CKD and healthy controls (HC) using 16S ribosomal DNA sequencing and examine its association with diabetes, hypertension, and immunity.

**Results:**

We observed that the bacterial community was skewed in the saliva of CKD, with increased *Lautropia* and *Pseudomonas*, and decreased *Actinomyces*, *Prevotella*, *Prevotella* 7, and *Trichococcus*. No difference in the bacterial community between the CKD patients complicated with and without diabetes, and between those with and without hypertension. *Prevotella* 7 declined in CKD patients with/without hypertension with respect to HC, while *Pseudomonas* increased in CKD patients with/without hypertension. *Pseudomonas* was negatively associated with immunoglobin G in CKD patients. Both CKD patients with positive and negative antistreptolysin O had declined *Prevotella* 7 and *Trichococcus* compared to HC, whereas increased *Pseudomonas*.

**Conclusions:**

Our study identifies a distinct bacterial saliva microbiome in CKD patients characterized by alteration in composition. We unravel here that the co-occurrence diseases of diabetes and hypertension are not associated with specific bacterial alterations, suggesting that bacterial dysbiosis in saliva plays a role in renal damage regardless of the occurrence of diabetes and hypertension.

**Supplementary Information:**

The online version contains supplementary material available at 10.1186/s12967-022-03602-5.

## Background

Chronic kidney disease (CKD) is a major and serious global health burden that leads to kidney failure as well as systemic diseases. Gut dysbiosis-a change in the microbial diversity in the gut-has been observed in CKD patients [1]. Likely, many studies have suggested that the microbiome in the oral cavity also plays an important role in the health of the host [2], and the association between the oral microbiome and CKD pathogenesis has been addressed. For example, Hu J et al. found that enrichment of *Neisseria* accompanied by depletion of *Veillonella* and *Streptococcus* in CKD patients [3]. Moreover, they detected a negative association between *Neisseria* and *Streptococcus* with the estimated glomerular filtration rate (eGFR) [3]. Duan X et al. reported that end-stage renal disease changeed the salivary microbiome in CKD patients, and it was associated with oral health state [4]. Notably, the periodontal pathogens were enriched in CKD patients undergoing hemodialysis [4]. Guo S et al. found that oral microbial diversity in CKD patients was increased. *Streptococcus*, *Actinomyces*, and *Leptotrichia* were enriched in CKD patients, while *Prevotella* and *Haemophilus* were decreased in CKD patients [5].

The potential impact of salivary microbiome on diabetes has been demonstrated in recent years. Several studies compared the salivary microbiome in patients with diabetes to that of healthy subjects using next generation sequence. Data showed that diabetes was associated with a decrease in bacterial diversity of the salivary microbiome [6–8]. In addition, higher salivary levels of *P. gingivalis*, *T. forsythia,* and *F. alocis* were reported in patients with gestational diabetes [9]. Although diabetic kidney disease develops in approximately 40% of patients who are diabetic and is the leading cause of CKD worldwide [10], and the association between the gut microbiome and diabetes has been extensively explored [11–13], few studies have reported the impact of the salivary microbiome on CKD patients complicated with diabetes.

As approximately 50% of adults had hypertension [14], the pathological role of the microbiome in hypertension, including the salivary microbiome, has gained increasing attention. Barbadoro P. et al. reported that bacterial species of dental pathologies, such as *Actinobacillus actinomycetemcomitans* had a higher concentration in an oral plaque of hypertension patients [15]. Gordon JH found that *Prevotella oral* and *Streptococcus oralis* increased in hypertension women taking antihypertension medications compared to those with normal blood pressure [16]. However, until now, the alteration of the salivary microbiome in hypertension patients with kidney damages has not been reported.

The kidneys are a frequent target of systemic immune and autoimmune disorders, including systemic autoimmunity and vasculitis, immune complex-related serum sickness, and complement disorders [17]. The human microbiome is responsible for interfacing with the induction, development, and modulation of immune responses [18]. Therefore, there might be biological interconnectivity of salivary microbiome and immunological profiles in CKD patients.

Here, we hypothesize that patients with CKD have a different microbial profile in saliva compared with healthy controls (HC). This deviation in the microbiome has a potential role in the etiology of the common diseases of diabetes and hypertension in CKD and the outcomes of their immunity.

## Materials and methods

### Study subjects

This study was approved by the Institutional Review Board of Wuxi Second Hospital of Nanjing Medical University (Ref. 2018051). All participants gave written informed consent before participation, and the study was carried out in accordance with the ethical standards in the Declaration of Helsinki. The inclusion criteria for the groups of CKD and HC were as follows: To be a participant,  ≥ 18 years, no antibiotic use for  ≥ 1 month before sampling, without acute intercurrent disease and infections/diarrhea/kidney transplantation/pregnancy/breastfeeding, have not been administered medications such as antibiotics/probiotic/immunosuppression within 1 month before enrollment. The diagnosis of CKD was based on the criteria: either kidney damage or Egfr  < 60 ml/min/1.73 m^2^ present for  ≥ 3 months. The markers of kidney damage include albuminuria [albumin excretion rate  ≥ 30 mg/24 h; urinary albumin creatinine ratio (UACR)  ≥ 30 mg/g], urine sediment abnormalities, electrolyte and other abnormalities due to tubular disorders, abnormalities detected by histology, and structural abnormalities detected by imaging [19]. Kidney damage refers to pathologic abnormalities or markers of damage, including abnormalities in blood or urine tests or imaging studies [20]. CKD patients in our present study had never undergone hemodialysis. Subjects with kidney damage (the markers of kidney damage as described above) or eGFR above 90 ml/min/1.73 m^2^, current illness including diabetes and hypertension, and acute or chronic infections, were excluded from the HC group.

We collected demographic and health status information through face-to-face interviews and medical review. Fasting blood glucose, HbA1c, and blood pressure were assessed on the day of recruitment. An immunoturbidimetric test was used to assess serum immunological parameters on the day of sample collection by Freelite test (AU5421; Beckman Coulter, USA).

To investigate the influence of co-occurrence of diabetes and hypertension in CKD on the salivary microbiome, we divided the CKD patients into subgroups of diabetic CKD (DM-CKD) and non-diabetic CKD (nonDM-CKD), and subgroups of hypertensive CKD (HTN-CKD) and non-hypertensive CKD (nonHTN-CKD).

### Sample collection, DNA isolation, and bioinformatic analysis

1 mL saliva was collected into an RNA-free sterile container after the participants refrained from eating, drinking, smoking, and brushing at least 1 h before saliva collection. The samples were added to 500 μL lysis buffer and stored at −80 ℃ until DNA isolation. The procedures of DNA extraction, 16S rRNA quantitative PCR, qualitative 16S targeted metagenomic sequencing, and bioinformatic analysis are described in our previous study [21]. In addition, a receiver operating characteristic (ROC) curve, was used to illustrate the diagnostic ability of bacterial genus. Linear discriminant analysis effect size (LefSe) was used to find biomarkers between groups. R studio (version 3.6.3) was used to perform the bioinformatics analysis. Functional metagenomes were predicted based on the 16S rRNA sequencing data of the salivary microbiome using PICRUSt2 (phylogenetic investigation of communities by reconstruction of unobserved states) v2.0.3 (https://github.com/picrust/picrust2) to predict functional gene abundance with Kyoto Encyclopedia of Genes and Genomes (KEGG) orthologs.

### Statistical analysis

Descriptive statistics for demographics and clinical and immunological parameters of groups are presented. To compare demographics and clinical characteristics between the groups, continuous variables were assessed using independent *t*-tests and categorical variables using Chi-square or Fisher’s exact tests, when appropriate. One-way analysis of variance was employed to compare quantitative variables among the number of groups  ≥ 3. The Wilcoxon rank-sum test/Kruskal–Wallis one-way test was applied to compare bacterial diversity and the relative abundance of bacterial taxa (≥ 1% of total abundance) between/among groups, and a Benjamini Hochberg false discovery rate (FDR)-corrected *P*-value was calculated for comparative tests. A *P* < 0.05 was used as a cut-off for comparative statistical tests. SPSS (v. 24.0) was used to compare the demographics between/among groups.

Next, we examined associations between the bacterial genus that exhibited a significant difference between the groups of CKD and HC and the levels of serum immunological profiles. For continuous variables, we used Pearson analysis, and a *P* < 0.05 was used as a significant cut-off correlation analysis. For categorical variables, such as anti-streptolysin O (ASO), we separated the CKD patients into subgroups of ASO positive CKD samples (ASOPOS-CKD) and ASO negative samples (ASONEG-CKD), and compared the bacterial microbiome among the groups of ASOPOS-CKD, ASONEG-CKD, and HC.

## Results

### Participants’ demographics and salivary microbiome characterization

A total of 200 salivary samples were analyzed, including 100 from CKD patients and 100 from HC (Table 1). The participants in the groups of CKD and HC were age-gender-BMI matched. In the CKD group, 29, 64 and 58 patients were with co-occurring type 2 diabetes mellitus (T2DM), hypertension, and detectable ASO respectively (Additional file 1: Table S1, Additional file 2: Table S2 and Additional file 3: Table S3). Among the 29 T2DM patients, 25 of them had hypertension, and 18 had detectable ASO; among the 64 hypertension patients, 25 of them had T2DM, and 35 of them had detectable ASO; among the 58 patients with detectable ASO, 18 of them had T2DM, and 35 had hypertension. As expected, the CKD group had a declined eGFR, and increased levels of serum urea, serum creatinine, serum uric acid, urine protein, urine creatinine, and blood pressure (*P* < 0.05). Also, the patient group had increased levels of serum C-reactive protein, κ FLC, and λ, FLC (*P* < 0.05), and decreased levels of C3 and C4. Among the CKD patients, ASOPOS subjects accounted for 58% (Additional file 3: Table S3).

**Table 1:** Characteristics of the participants

Parameters	CKD (n = 100)	HC (n = 100)	*P* value
Age (yr)	58.18 ± 15.53	60.84 ± 15.36	0.300
Duration (yr)	3.96 ± 4.17	NA	NA
Men (n%)	40 (40.00)	40 (40.00)	1.000
Body mass index (kg/m^2^)	24.96 ± 3.90	24.84 ± 2.59	0.827
Co-current disease (n%)			
Type 2 diabetes mellitus	29 (29.00)	0 (0.00)	< 0.001
Hypertension	64 (64.00)	0 (0.00)	< 0.001
eGFR (mL/min/1.73m^2^)	53.44 ± 40.32	104.57 ± 17.90	< 0.001
Serum urea (mmol/L)	13.39 ± 11.39	5.41 ± 1.65	< 0.001
Serum creatinine (mg/dL)	216.91 ± 206.60	60.19 ± 12.26	< 0.001
Serum uric acid (umol/L)	425.56 ± 132.15	294.79 ± 89.35	< 0.001
Urine protein (n%)			< 0.001
Negative	48 (48.00)	100 (0.00)	
Positive 1 plus	23 (23.00)	0 (0.00)	
Positive 2 plus	21 (21.00)	0 (0.00)	
Positive 3 plus	8 (8.00)	0 (0.00)	
Urine creatinine (mmol/L)	6.61 ± 2.86	4.40 ± 1.91	< 0.001
24 h urine protein (mg/dL)	2797.04 ± 2474.04	2664.90 ± 255.98	0.867
HbA1c (%)	6.26 ± 1.23	6.29 ± 0.60	0.858
FBG (mmol/L)	5.95 ± 3.21	5.76 ± 2.23	0.691
Systolic blood pressure (mmHg)	148.91 ± 22.48	128.52 ± 14.01	< 0.001
Diastolic blood pressure (mmHg)	84.54 ± 12.84	77.53 ± 9.04	< 0.001
Ig A (g/L)	2.59 ± 1.12	2.47 ± 0.82	0.462
Ig G (g/L)	10.93 ± 3.80	10.98 ± 1.40	0.652
Ig M (g/L)	1.21 ± 0.95	1.18 ± 0.53	0.982
C-reactive protein (mg/L)	6.63 ± 11.11	2.36 ± 3.10	0.003
Completement 3 (g/L)	0.81 ± 0.23	0.98 ± 0.16	< 0.001
Completement 4 (g/L)	0.19 ± 0.03	0.24 ± 0.08	< 0.001
Serum κ FLC (g/L)	8.93 ± 3.12	3.09 ± 0.72	< 0.001
Serum λ FLC (g/L)	4.70 ± 1.50	1.77 ± 0.46	< 0.001
ASO			< 0.001
ASOPOS (n%)	58 (58.00)	0 (0 .00)	
ASONEG (n%)	42 (42.00)	100 (100.00)	

Pearson’s Chi-square/Fisher’s exact test was used to compare dichotomous variables, and an independent *t*-test was used to compare continuous variables

*ASONEG* antistreptolysin O negative, *ASOPOS* antistreptolysin O positive, *CKD* chronic kidney disease, *eGFR* estimated glomerular filtration rate, *FBG* fasting blood glucose, *FLC* free light chains, *HbA1c* hemoglobin A1c

After excluding samples with invisible bands in PCR, we characterized the bacterial composition of salivary samples from 198 subjects, including 98 diagnosed with CKD and 100 healthy subjects. A total of 16,504,359 raw reads were identified, after removing low-quality or ambiguous reads 14,869,458 valid reads have remained. After rarifying to 15,193 reads, we obtained 10,388 ASVs. Good’s coverage index in each sample had a value of 100% measured at the amplicon sequence variant (ASV) level.

### Bacterial community and diversity in CKD

**Fig. 1:**
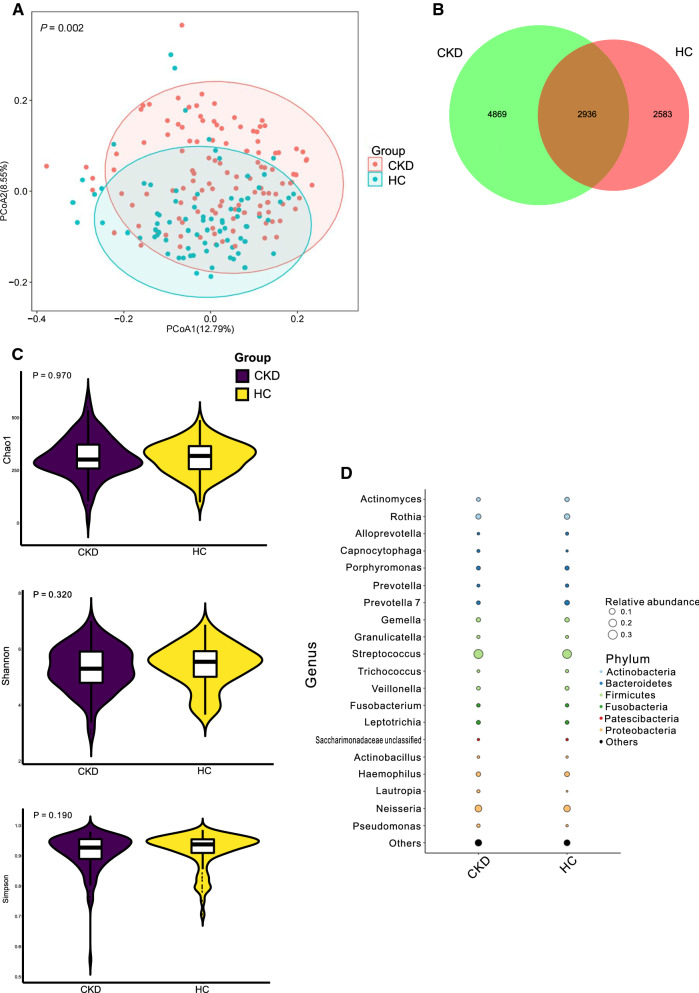
Microbial community, diversity, and composition **A**. PCoA based on Bray–Curtis distances at ASV level showed different microbial compositions between groups of CKD patients and HCs (*P* < 0.05). Permutational multivariate analysis of variance (PERMANOVA) was performed for statistical comparisons of samples in the two cohorts. *P-value* was adjusted by the Benjamini and Hochberg false discovery rate (FDR). **B**. Venn diagram showing a dissimilar number of ASVs shared by the groups of CKD and HC. **C**. Bacterial richness and diversity measured by Chao1, Shannon, and Simpson were calculated at the microbial ASV level. Wilcoxon rank-sum test was performed and adjusted by Benjamini and Hochberg false discovery rate (FDR). **D**. Microbial profile at the phylum and genus level. Bubble plot showing the abundances of bacterial taxa as a percentage of the entire bacteria population detected in the groups of CKD and HC. The taxonomic classification levels of phylum and genus are displayed. The relative abundance of a genus in a group is scaled to match the size of the corresponding point

PCoA for the significantly differential ASVs indicated that the bacterial community in CKD patients’ saliva was different from that in the HC group (*P* < 0.05; Fig. 1A). Of 10,388 observed ASVs, 28.26% of them was shared by the two groups (Fig. 1B). However, when the bacterial richness and diversity were compared, the index of Chao 1, Shannon, and Simpson did not show a difference between the groups of CKD and HC (*P* > 0.05; Fig. 1C).

### Bacterial composition in CKD

As Fig. 1D shown, the most abundant bacterial phylum in both CKD and HC groups were Firmicutes (44.25% in CKD and 43.38% in HC) and its genus *Streptococcus* (30.50% in CKD and 28.45% in HC). Firmicutes was followed by Proteobacteria (26.61% in CKD and 22.40% in HC), and its bacterial genus such as *Neisseria* was accounted as the second most abundant bacteria in both CKD and HC (14.00% and 12.94%, respectively). Actinobacteria exhibited a decline in the CKD group (10.77%) compared to HC (13.77%). Also, *Rothia*, a bacterial genus belonging to Actinobacteria, showed a decreasing trend in the CKD (6.99%) relative to HC (8.37%).

### Alterations of a bacterial taxon in CKD

**Fig. 2:**
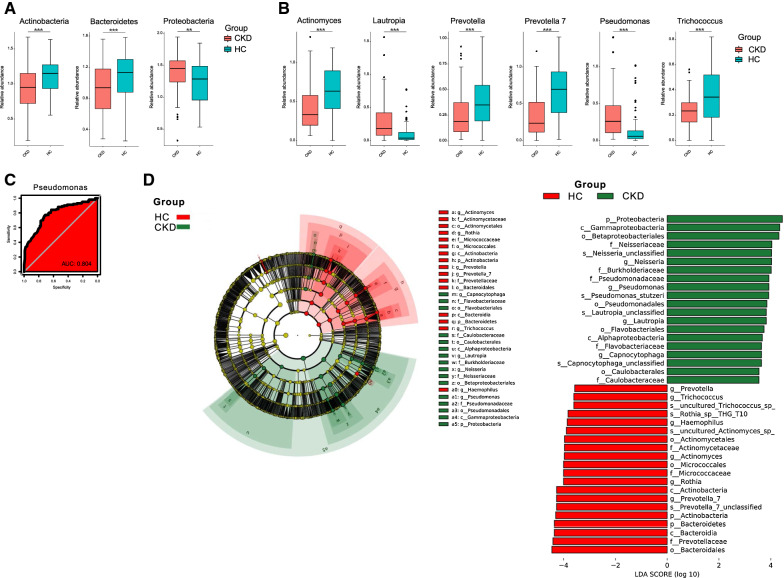
Bacterial taxon comparisons **A**. Microbial phylum was differentially abundant between CKD patients and HCs. Only the phylum with above 1% is displayed. *P-value* was calculated using Wilcoxon rank-sum test and adjusted by Benjamini and Hochberg’s false discovery rate (FDR). ** and *** indicate *P* < 0.01 and *P* < 0.001, respectively. **B**. Microbial genus was differentially abundant between CKD patients and HCs. Only the phylum with above 1% is displayed. *P-value* was calculated using Wilcoxon rank-sum test and adjusted by Benjamini and Hochberg’s false discovery rate (FDR). ** and *** indicate *P* < 0.01 and *P* < 0.001, respectively. **C**. The area under the ROC curve (AUC) of salivary microbiome based on CKD classification. Random forest classifiers were sued to separate CKD patients and HC based on ASV-level composition. The red area represents the 95% confidence interval shape. **D**. Linear Discriminant Analysis (LDA) Effect Size (LEfSe) plot of taxonomic biomarkers identified in the salivary microbiome of participants. Specific bacterial traits are found at all taxon levels. The threshold for the logarithmic discriminant analysis (LDA) score was 3

When the bacterial phylum was compared between the groups of CKD and HC. Significantly decreased abundances of Actinobacteria and Bacteroidetes were observed in CKD group, whereas Proteobacteria increased in CKD (P < 0.05; Fig. 2A).

When the Wilcoxon rank-sum test was applied, the bacterial genus of *Actinomyces*, *Prevotella*, *Provotella* 7, and *Trichococcus* significantly decreased in CKD than those in HC, while *Lautropia* and *Pseudomonas* increased in CKD (*P* < 0.05; Fig. 2B). ROC measured by AUC showed that *Pseudomonas* had an AUC value of 0.804 (Fig. 2C). LefSe showed that Proteobacteria and its genus *Pseudomonas* can be classified as CKD biomarkers, while *Prevotella*, *Prevotella* 7, and *Trichococcus* can be considered healthy biomarkers in saliva (Fig. 2D).

### Alterations of functional pathway

**Fig. 3:**
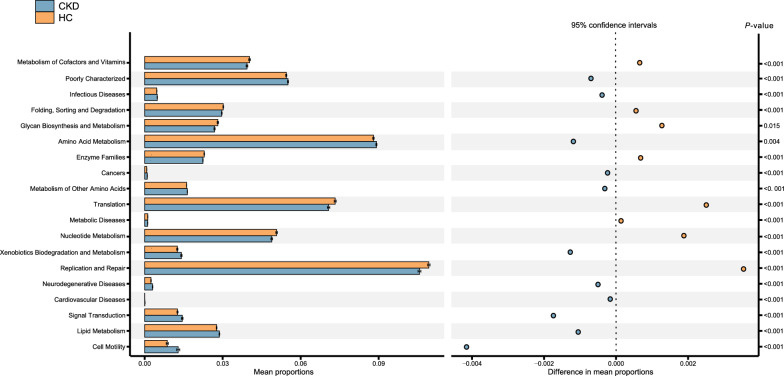
Prediction of altered KEGG pathways using PICRUSt2 analysis. Bar plots on the left side display the mean proportion of each KEGG pathway. Dot plots on the right show the differences in mean proportions between the two indicated groups. *P-value* was calculated using Wilcoxon rank-sum test and adjusted by Benjamini and Hochberg’s false discovery rate (FDR)

In order to determine the functional implication of microbial composition in CKD, we used PCIRUSt2 to infer microbial gene content from 16S rRNA gene data and aggregated relative abundance of functional genes into metabolic pathways. CKD salivary samples exhibited significantly higher levels of amino acid metabolism, lipid metabolism, xenobiotics biodegradation and metabolism, etc., whereas had lower levels of metabolism of cofactors and vitamins, glycan biosynthesis and metabolism, cardiovascular diseases, etc.(P < 0.05; Fig. 3).

### Alterations of the salivary microbiome in DM-CKD and HTN-CKD patients

**Fig. 4:**
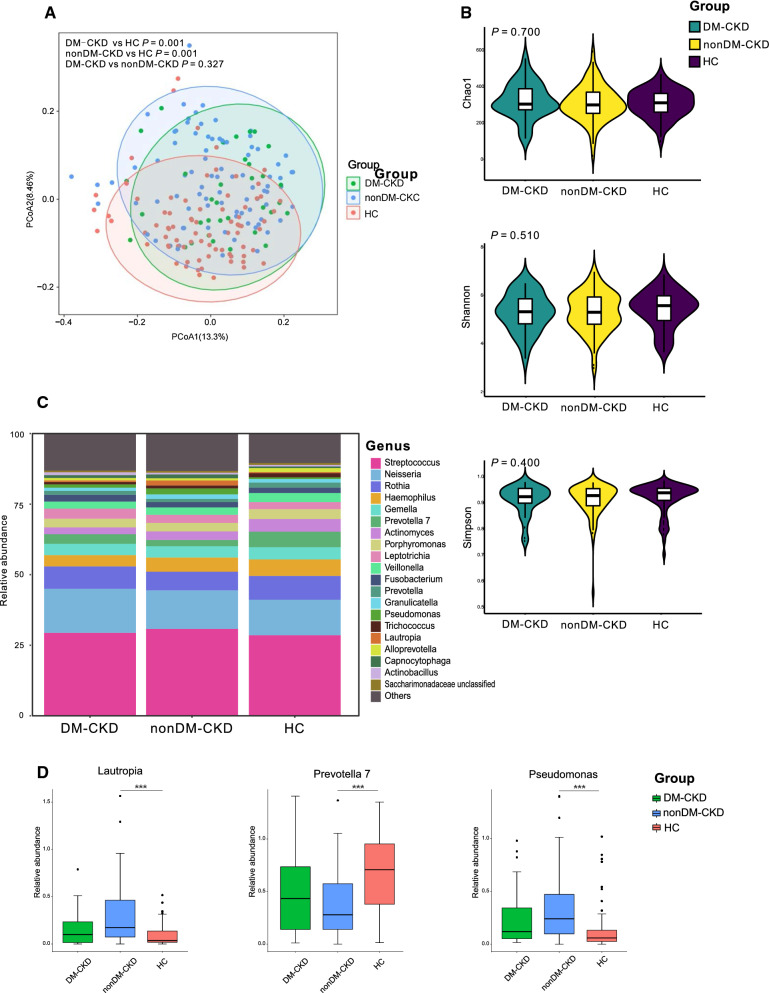
Association of diabetes in the salivary microbiome in CKD **A**. PCoA based on Bray–Curtis distances at ASV level showed different microbial compositions between groups of DM-CKD patients and HC (*P* < 0.05); between nonDM-CKD patients and HC(*P* < 0.05); between DM-CKD patients and nonDM-CKD patients (*P* > 0.05). Permutational multivariate analysis of variance (PERMANOVA) was performed for statistical comparisons of samples in the two cohorts. *P-value* was adjusted by the Benjamini and Hochberg false discovery rate (FDR). **B**. Bacterial richness and diversity measured by Chao1, Shannon, and Simpson were calculated at the microbial ASV level. Wilcoxon rank-sum test was performed and adjusted by Benjamini and Hochberg false discovery rate (FDR). **C**. Microbial profile at the genus level. The stacked plot demonstrated the top 15 most abundance bacterial genera in the groups of HC, DM-CKD, and nonDM-CKD. **D**. Bacterial genus that was differentially abundant among groups of HC, DM-CKD, and nonDM-CKD. Only the genus with above 1% is displayed. *P-value* was calculated using Wilcoxon rank-sum test and adjusted by Benjamini and Hochberg’s false discovery rate (FDR). *** indicate *P* < 0.001

When we separated the CKD patients into subgroups of DM-CKD and nonDM-CKD, both of them displayed significantly different bacterial communities from that in HC (*P* < 0.05; Fig. 4A), whereas no difference between DM-CKD and nonDM-CKD (*P* > 0.05; Fig. 4A). The bacterial richness and diversity, including indices of Chao 1, Shannon, and Simpson did not show significant differences among the groups of DM-CKD, nonDM-CKD, and HC (*P* > 0.05; Fig. 4B). The top 5 abundant bacterial genera were *Streptococcus*, *Neisseria*, *Rothia*, *Gemella,* and *Haemophilus* in the DM-CKD group; and the most abundant bacterial genera in the nonDM-CKD group were the same as the DM-CKD group; *Streptococcus*, *Neisseria*, *Rothia* and *Haemophilus* were also accounted for the most abundant bacteria in the HC group, and *Prevotella* 7 was stand out in the HC (Fig. 4D). Surprisingly, *Lautropia* and *Pseudomonas* only showed a decline in group HC compared to the nonDM-CKD group instead of the DM-CKD group (*P* < 0.05; Fig. 4D). Similarly, *Prevotella* 7 only increased in the HC group with respect to the nonDM-CKD instead of the DM-CKD group (*P* < 0.05; Fig. 4D).

**Fig. 5:**
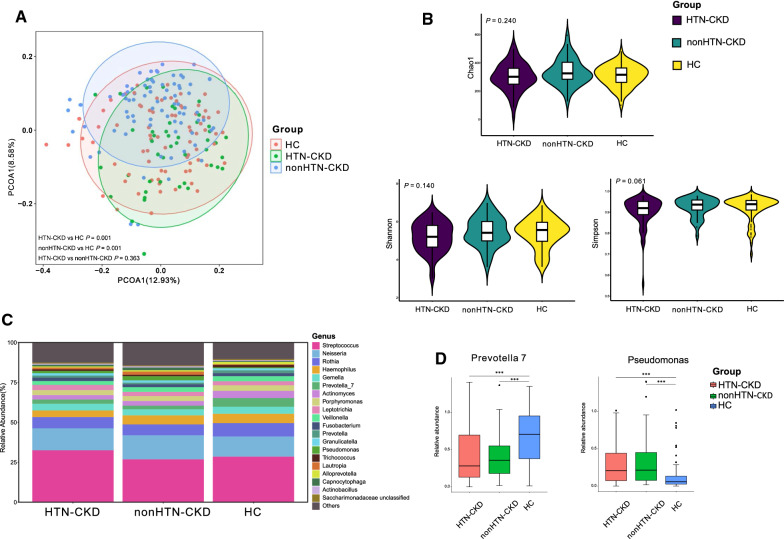
Association of hypertension in the salivary microbiome in CKD **A**. PCoA based on Bray–Curtis distances at ASV level showed different microbial compositions between groups of HTN-CKD patients and HC (*P* < 0.05); between nonHTN-CKD patients and HC (*P* < 0.05); between HTN-CKD patients and nonHTN-CKD patients (*P* > 0.05). Permutational multivariate analysis of variance (PERMANOVA) was performed for statistical comparisons of samples in the two cohorts. *P-value* was adjusted by the Benjamini and Hochberg false discovery rate (FDR). **B**. Bacterial richness and diversity measured by Chao1, Shannon, and Simpson were calculated at the microbial ASV level. Wilcoxon rank-sum test was performed and adjusted by Benjamini and Hochberg false discovery rate (FDR). **C**. Microbial profile at the genus level. The stacked plot demonstrated the top 15 most abundantbacterial genera in the groups of HC, HTN-CKD, and none-CKD. **D**. Bacterial genus that was differentially abundant among groups of HC, HTN-CKD, and nonHTN-CKD. Only the genus with above 1% is displayed. *P-value* was calculated using Wilcoxon rank-sum test and adjusted by Benjamini and Hochberg’s false discovery rate (FDR). *** indicates *P* < 0.001

Similar to the alteration trend in DM-CKD, both the groups of HTN-CKD and none-CKD displayed a significant difference in the bacterial community from the group of HC (*P* < 0.05; Fig. 5A), while the group of HNT-CKD did not differ from nonHTN-CKD (*P* > 0.05; Fig. 5A). Also, the bacterial richness and diversity were not different among the groups of HTN-CKD, nonHTN-CKD, and HC (*P* > 0.05; Fig. 5B). Figure 5C displayed that the sequence of the most five abundant bacterial genera in the group of HTN-CKD was *Streptococcus*, *Neisseria*, *Rothia*, *Gemella,* and *Haemophilus*, and the most abundant bacterial genus in the nonHTN-CKD group was ranked as *Streptococcus*, *Neisseria*, *Rothia*, *Haemophilus* and *Gemella*. When the bacterial genus was compared, both HTN-CKD and the nonHTN-CKD group had a significantly lower level of *Prevotella* 7 than group HC. In contrast, both of them had a higher level of *Pseudomonas* than group HC (*P* < 0.05; Fig. 5D).

### Associations of the salivary microbiome with immunity

**Fig. 6:**
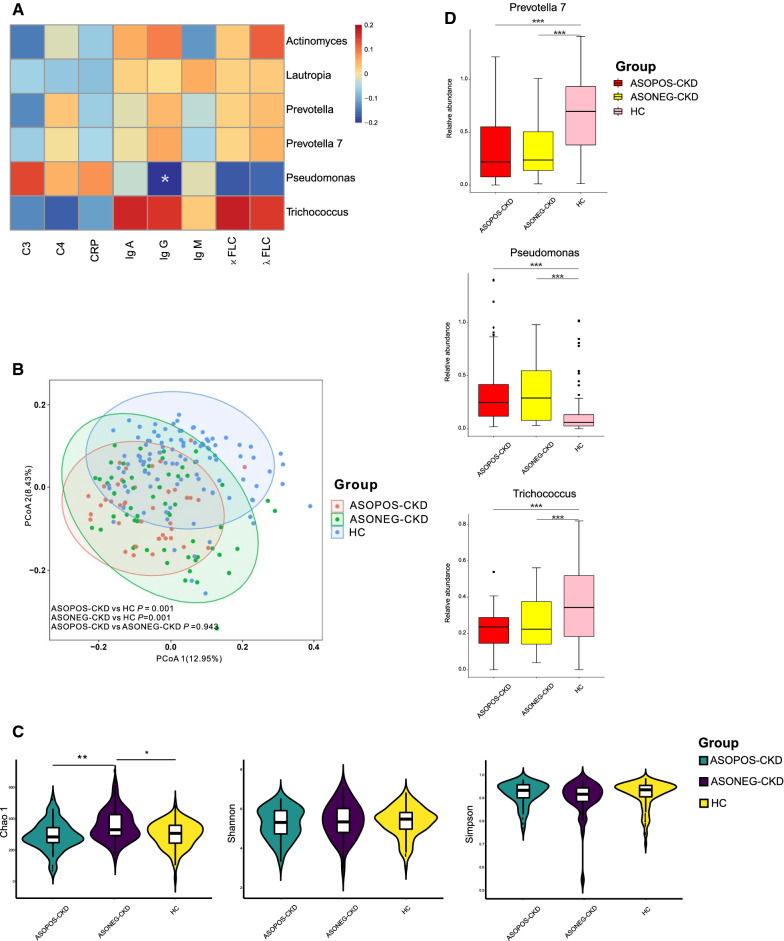
Association of the salivary microbiome with immunity **A**. The heatmap depicted the association between bacterial genera which differed in CKD relative to HC and the value of immunological profiles in CKD patients. Pearman correlation analysis was performed. * indicates *P* < 0.05. **B**. PCoA based on Bray–Curtis distances at ASV level showed different microbial compositions between groups of ASOPOS-CKD patients and HC (*P* < 0.05); between ASONEG-CKD patients and HC (*P* < 0.05); between ASOPOS-CKD patients and ASONEG-CKD patients (*P* > 0.05). Permutational multivariate analysis of variance (PERMANOVA) was performed for statistical comparisons of samples in the two cohorts. *P-value* was adjusted by the Benjamini and Hochberg false discovery rate (FDR). **C**. Bacterial richness and diversity measured by Chao1, Shannon, and Simpson were calculated at the microbial ASV level. Wilcoxon rank-sum test was performed and adjusted by Benjamini and Hochberg false discovery rate (FDR). * and ** indicate *P* < 0.05 and *P* < 0.01, respectively. D. Bacterial genus that was differentially abundant among groups of HC, ASOPOS-CKD, and ASONEG-CKD. Only the genus with above 1% is displayed. *P-value* was calculated using Wilcoxon rank-sum test and adjusted by Benjamini and Hochberg’s false discovery rate (FDR). *** indicates *P* < 0.001

To seek the association between bacterial microbiome and immunity, we performed Pearson correlation analysis between the bacterial genus showing significantly increased or decreased in CKD and the parameters of immunological indicators in CKD and noticed that *Pseudomonas* was significantly correlated to the level of serum IgG (*P* < 0.05; Fig. 6A). When the CKD patients were separated into subgroups of ASOPOS-CKD and ASONEG-CKD, both of them showed a different bacterial community from HC, whereas no difference was observed between the subgroups of ASOPOS-CKD and ASONEG-CKD (*P* < 0.05; Fig. 6B). ASONEG-CKD patients had a significantly higher level of bacterial richness of Chao 1 compared to ASOPOS-CKD patients and HC (*P* < 0.05; Fig. 6C); both *Prevotella* 7 and *Trichococcus* showed significantly lower levels in groups of ASOPOS-CKD and ASONEG-CKD comparing to HC, while *Pseudomonas* enriched in ASOPOS-CKD and ASONEG-CKD comparing to HC (*P* < 0.05; Fig. 6D).

## Discussion

Following a large number of studies on the human gut microbiome in an unhealthy state, the role of the salivary microbiome in health has been gradually explored [2]. As a study that is not evenly matched with age-gender-BMI between cases and controls, confound microbiome analyses can produce spurious microbial associations with human diseases [22], we performed an age-gender-BMI matched case–control study to examine the salivary microbiome in non-dialysis CKD patients. In addition, our present study assessed the associations of co-occurrence of diabetes and hypertension and immunity which has been rarely reported.

We observed a distinct bacterial community in the saliva of CKD patients. Such an alteration in CKD patients was also reported by previous studies [3, 5]. In addition, we noticed that no bacterial richness and diversity changes were observed in CKD patients, which were dissimilar to previous studies [4]. A higher level of bacterial diversity in CKD patients’ saliva has been reported by both Duan X et al. and Guo S. et al. on the Chinese population [4, 5]. However, no consistent findings of alteration trend in bacterial richness in CKD patients were demonstrated by their studies. Based on the two previous studies and our present study, it is hard to conclude that there is an association between bacterial richness and diversity in the salivary microbiome in the Chinese CKD population.

As diabetes and hypertension play vital importance in the progression of kidney damage [23], we assessed the salivary microbiome in CKD patients with and without diabetes/hypertension. Although both of DM/HTN-CKD and nonDM/HTN-CKD patients demonstrated a different bacterial community and bacterial diversity from those in the controls, no differences were observed between those with and without complications of diabetes/hypertension. A similar finding was reported by a human gut microbiome study [24]. In their study, Tao S et al. also noticed that there was no difference between CKD patients with and without diabetes in their microbial community and diversity [24]. Under this perspective, although the relationship between kidney damage and salivary microbiome can be defined, the specific contributors of diabetic and hypertensive nephropathy for the profile of microbiome should be explored using more clinical and animal studies.

It is worthy to note, although the ASOPOS-CKD and ASONEG-CKD patients did not show difference in the bacterial community, the ASONEG-CKD patients had higher bacterial richness than the ASOPOS-CKD patients. The association between the salivary microbiome and anti-streptolysin O has never been reported, whereas its association with the gut microbiome revealed a negative correlation with *Lactobacillus* the concentration of anti-streptolysin O titer [25], which indicates that positive ASO suppresses the growth of probiotic bacteria, such as *Lactobacillus*.

There were several bacterial taxa exhibiting alterations that have never been reported by previous salivary studies on CKD, such as bacterial phylum Actinobacteria and its genus *Actinomyces* sharply depleted in CKD patients. This finding is not consistent with Guo S. et al. report. In their study, *Actinomyces* increased in the CKD group [5].

*Prevotella*, as one of the major bacterial genera in the oral microbiome [26], is depleted in CKD patients. Comparisons of bacterial genus among the three groups of HTN-CKD, nonHTN-CKD, and HC demonstrated that *Prevotella* 7 plays a role in CKD, regardless of the complication of hypertension. *Prevotella* spp., a proteolytic/amino acid–degrading bacteria, can break down proteins and peptides into amino acids and degrade them further via specific pathways to produce short-chain fatty acids [27], and its species are considered commensal microbes in the oral cavity [28]. However, several recent studies demonstrated that *Prevotella* is associated with diseases, such as periapical infection [29], gout [30], and multiple sclerosis [31]. Thus, although *Prevotella* declined in CKD patients which included those with and without hypertension, its role needs further investigation by salivary microbiome transplantation in CKD patients and animal models.

The enrichment of *Pseudomonas* in CKD patients is outstanding in our present study, as it was confirmed by Wilcoxon signed-rank test, ROC curve, and LefSe analysis. In addition, the abundance of *Pseudomonas* declined in both the CKD patients complicated with and without hypertension when compared to controls. We noticed that the level of immunoglobulin G (IgG) antibody was negatively associated with the abundance of *Pseudomonas*. IgG is a major component of humoral immunity [32]. According to data from the literature, lower serum IgG level has been associated with a higher proportion of chronic pathological changes, lower eGFR, and poor renal outcome [33]. The negative association between Ig G and *Pseudomonas* indicates that the salivary microbiome plays a role in regulating CKD patients’ immunity.

We observed that *Lautropia* increased in CKD patients. Enrichment of salivary *Lautropia* might indicate an unhealthy state in the human oral cavity, as several previous studies demonstrated that *Lautropia* increased in various diseases. For example, Yu F and his group reported that oral lichen planus patients with erosive lesions had a higher level of *Lautropia* than those without erosive lesions and healthy subjects [34]. Snider E J et al. found that *Lautropia* can be listed as a diagnostic biomarker for patients with Barrett’s esophagus [35]. Also, Li D et al. reported that *Lautropia* could be used for the diagnosis of hepatitis B patients [36].

The depletion of *Trichococcus* in CKD patients might be associated with an unhealthy state in the oral cavity. A previous study also demonstrated a decrease of *Trichococcus* in pediatric patients with obstructive sleep apnea [37]. A further investigation of the function of *Trichococcus* using animal models is necessary.

When comparing the metabolic pathway data of the salivary microbiome, we found several metabolic pathways were associated with CKD. The upregulation of the pathway of lipid metabolism is inconsistent with a previous oral study on CKD patients [3], while the downregulation of metabolism of cofactors and vitamins is similar to a gut study on CKD [38]. A further study using metabolome identification is needed for exploring the metabolism in CKD and its association with salivary microbiome.

Like previous studies on the human salivary microbiome[4, 5], our present study again demonstrated that CKD patients had a distinct microbiome from controls. However, the complications of diabetes and hypertension act out in the microbial community in CKD patients. As saliva is a diagnostic fluid with easy and non-invasive collection, the potential value of *Pseudomonas* as a biomarker of CKD in saliva suggests that we should make detailed investigations in clinical settings.

Although therapeutic interventions aimed at restoring bacterial flora in the saliva in CKD patients may be future targets, current salivary microbiome study in a single-center is limited, necessitating further research. Further multi-center studies correlating the salivary microbiome with the gut microbiome, intestinal permeability markers, inflammatory markers, epigenetic factors, and various etiologies of CKD are needed to better interpret the salivary microbiome as a potential diagnostic biomarker and to investigate the diagnostic value and therapeutic effect in CKD patients. When the diagnostic value is confirmed by several large and multi-center studies, salivary microbiome transplantation should be investigated to replace the treatment option of fecal microbiome transplantation for CKD. As saliva is more easily collected using sterile collection tubes from healthy donors comparing to fecal samples.

Our present study has some limitations. First, only local community participants were involved in this study. Human microbiome studies demonstrated that sampling a broad population of humans representing different cultural traditions offers an opportunity to discover how our microbiomes vary between populations [39]. Thus, multicenter studies in geographically distinct areas with more study participants, as well as longitudinal study designs that consider individual differences in the salivary microbiomes of CKD individuals, are essential for a dysbiosis of this prevalence. Second, although it seems that the complications of diabetes and hypertension were not associated with the alteration of salivary microbiome in CKD patients, the sample size in the subgroups of DM-CKD/HTN-CKD and nonDM-CKD/nonHTN-CKD were too small and unequal, which might lead to statistical bias. A further study with larger and equal sample size design is necessary to re-assess the findings of the present study.

## Conclusions

In our study, we identified a distinct bacterial saliva microbiome in CKD patients characterized by alteration in composition. Meanwhile, we unravel here that the co-occurrence diseases of diabetes and hypertension are not associated with specific bacterial alterations, suggesting that bacterial dysbiosis in saliva plays a role in renal damage regardless of the occurrence of diabetes and hypertension.

## Supplementary Information

**Additional file 1:** **Table S1.** Characteristics of groups of DM-CKD, nonDM-CKD and HC.

**Additional file 2:** **Table S2.** Characteristics of groups of HTN-CKD, nonHTN-CKD and HC

**Additional file 3:** **Table S3.** Characteristics of groups of ASOPOS-CKD, ASONEG-CKD and HC

## Data Availability

16S rRNA sequencing information has been deposited into National Center for Biotechnology Information (NCBI) Sequence Read Archive (SRA) with reference PRJNA835723 (https://www.ncbi.nlm.nih.gov/sra/PRJNA835723).

## Abbreviations

CKD: Chronic kidney disease
GFR: Estimated glomerular filtration rate
HTN: Hypertension
HC: Healthy controls
UACR: Urinary albumin creatinine ratio
ROC: Receiver operating characteristic
ASO: Anti-streptolysin O
