# Supplementary material for: Salivary microbiome in chronic kidney disease: what is its connection to diabetes, hypertension, and immunity?
Source: J Transl Med. 2022 Sep 4;20:387. doi: 10.1186/s12967-022-03602-5 (PMC9441058; doi:10.1186/s12967-022-03602-5)
Supplement: Supplementary file 1 — Additional file 1: Table S1. Characteristics of groups of DM-CKD, nonDM-CKD and HC. [file 12967_2022_3602_MOESM1_ESM.doc]

**Table S1 Characteristics of groups of DM-CKD, nonDM-CKD and HC**

| **Parameters** | **DM-CKD (n = 29)** | **nonDM-CKD (n = 71)** | **HC (n = 100)** | ***P* value** |
| --- | --- | --- | --- | --- |
| Age (yr) | 61.72 ± 10.77 | 56.54 ± 17.14 | 60.84 ± 15.36 | 0.224 |
| Duration (yr) | 5.00 ± 4.63 | 3.48 ± 3.89 | NA | NA |
| Men (n%) | 12 (41.38) | 28 (39.44） | 40 (40) | 0.984 |
| Body mass index (kg/m^2^) | 25.64 ± 3.65 | 24.64 ± 4.01 | 24.84 ± 2.59 | 0.464 |
| eGFR (mL/min/1.73m^2^) | 58.45 ± 40.61 | 42.62 ± 38.24 | 104.57 ± 17.90 | < 0.001 |
| Serum urea (mmol/L) | 15.95 ± 10.67 | 12.20 ± 10.67 | 5.41 ± 1.65 | < 0.001 |
| Serum creatinine (mg/dL) | 251.21 ± 197.90 | 201.03 ± 210.41 | 60.19 ± 12.26 | < 0.001 |
| Serum uric acid (umol/L) | 447.62 ± 116.42 | 415.35 ± 138.66 | 294.79 ± 89.35 | < 0.001 |
| HbA1c (%) | 7.18 ± 1.44 | 5.84 ± 0.84 | 6.29 ± 0.60 | < 0.001 |
| FBG (mmol/L) | 7.37 ± 5.27 | 5.29 ± 1.11 | 5.76 ± 2.23 | 0.007 |
| Urine creatinine (mmol/L) | 5.88 ± 1.90 | 6.96 ± 3.16 | 4.40 ± 1.91 | < 0.001 |
| 24 h urine protein (mg/dL) | 2949.30 ± 2301.65 | 2726.56 ± 2567.72 | 2664.90 ± 255.98 | 0.914 |

Pearson’s Chi-square/Fisher’s exact test was used to compare dichotomous variables, and an independent *t*-test was used to compare continuous variables.

Abbrevation: CKD: chronic kidney disease; DM: diabetes mellitus; eGFR: estimated glomerular filtration rate; FBG: fasting blood glucose; HbA1c: hemoglobin A1c
