# Supplementary material for: Salivary microbiome in chronic kidney disease: what is its connection to diabetes, hypertension, and immunity?
Source: J Transl Med. 2022 Sep 4;20:387. doi: 10.1186/s12967-022-03602-5 (PMC9441058; doi:10.1186/s12967-022-03602-5)
Supplement: Supplementary file 2 — Additional file 2: Table S2. Characteristics of groups of HTN-CKD, nonHTN-CKD and HC [file 12967_2022_3602_MOESM2_ESM.doc]

**Table S2 Characteristics of groups of HTN-CKD, nonHTN-CKD and HC**

| **Parameters** | **HTN-CKD (n = 64)** | **nonHTN-CKD (n = 36)** | **HC (n = 100)** | ***P* value** |
| --- | --- | --- | --- | --- |
| Age (yr) | 55.46 ± 16.56 | 59.67 ± 14.90 | 60.84 ± 15.36 | 0.300 |
| Duration (yr) | 4.37 ± 4.51 | 3.21 ± 3.42 | NA | NA |
| Men (%) | 13 (20.31) | 28 (77.78） | 40 (40) | <0.001 |
| Body mass index (kg/m^2^) | 25.46± 3.77 | 24.04 ± 4.04 | 24.84 ± 2.59 | 0.191 |
| eGFR (mL/min/1.73m^2^) | 38.48 ± 31.15 | 80.70 ± 41.31 | 104.57 ± 17.90 | < 0.001 |
| Serum urea (mmol/L) | 16.66 ± 12.59 | 7.43 ± 4.93 | 5.41 ± 1.65 | < 0.001 |
| Serum creatinine (mg/dL) | 273.44 ± 224.81 | 113.94 ± 112.63 | 60.19 ± 12.26 | < 0.001 |
| Serum uric acid (umol/L) | 458.22 ± 136.78 | 366.08 ± 100.59 | 294.79 ± 89.35 | < 0.001 |
| Systolic blood pressure (mmHg) | 152.67 ± 24.50 | 142.07 ± 16.51 | 128.53 ±14.01 | < 0.001 |
| Diastolic blood pressure (mmHg) | 85.80 ± 13.06 | 82.25 ± 12.34 | 77.52 ± 9.04 | < 0.001 |
| Urine creatinine (mmol/L) | 6.36 ± 2.55 | 7.09 ± 3.34 | 4.40 ± 1.91 | < 0.001 |
| 24 h urine protein (mg/dL) | 2687.96 ± 2255.12 | 2995.71 ± 2863.82 | 2664.90 ± 255.98 | 0.845 |

Pearson’s Chi-square/Fisher’s exact test was used to compare dichotomous variables, and an independent *t*-test was used to compare continuous variables.

Abbrevation: CKD: chronic kidney disease; eGFR: estimated glomerular filtration rate; HTN: hypertension
