# Supplementary material for: Salivary microbiome in chronic kidney disease: what is its connection to diabetes, hypertension, and immunity?
Source: J Transl Med. 2022 Sep 4;20:387. doi: 10.1186/s12967-022-03602-5 (PMC9441058; doi:10.1186/s12967-022-03602-5)
Supplement: Supplementary file 3 — Additional file 3: Table S3. Characteristics of groups of ASOPOS-CKD, ASONEG-CKD and HC [file 12967_2022_3602_MOESM3_ESM.doc]

**Table S3 Characteristics of groups of ASOPOS-CKD, ASONEG-CKD, and HC**

| **Parameters** | **ASOPOS-CKD (n = 58)** | **ASONEG-CKD (n = 42)** | **HC (n = 100)** | ***P* value** |
| --- | --- | --- | --- | --- |
| Age (yr) | 57.74 ± 14.86 | 58.81 ± 16.69 | 60.84 ± 15.36 | 0.559 |
| Duration (yr) | 3.81 ± 4.30 | 4.19 ± 4.04 | NA | NA |
| Men (n%) | 15 (25.86) | 25 (59.52） | 40 (40) | 0.003 |
| Body mass index (kg/m^2^) | 25.10 ± 4.42 | 24.76 ± 3.05 | 24.84 ± 2.59 | 0.889 |
| eGFR (mL/min/1.73m^2^) | 58.13 ± 39.34 | 46.56 ± 41.37 | 104.57 ± 17.90 | < 0.001 |
| Serum urea (mmol/L) | 16.72 ± 14.29 | 11.11 ± 8.33 | 5.41 ± 1.65 | < 0.001 |
| Serum creatinine (mg/dL) | 176.88 ± 158.86 | 275.71 ± 252.89 | 60.19 ± 12.26 | < 0.001 |
| Serum uric acid (umol/L) | 408.94 ± 138.91 | 449.98 ± 119.46 | 294.79 ± 89.35 | < 0.001 |
| Urine creatinine (mmol/L) | 6.87 ± 2.89 | 6.25 ± 2.82 | 4.40 ± 1.91 | < 0.001 |
| 24 h urine protein (mg/dL) | 3105.69 ± 2866.12 | 2343.72 ± 1689.66 | 2664.90 ± 255.98 | 0.361 |

Pearson’s Chi-square/Fisher’s exact test was used to compare dichotomous variables, and an independent *t*-test was used to compare continuous variables.

Abbrevation: ASONEG: antistreptolysin O negative; ASOPOS: antistreptolysin O positive; CKD: chronic kidney disease; eGFR: estimated glomerular filtration rate
